# Health System Community Partnership to Design an Online Resource to Address Perinatal Information Needs for Black Families: Action Research Study

**DOI:** 10.2196/71161

**Published:** 2026-02-24

**Authors:** Yhenneko J Taylor, Alicia Dahl, McKenzie Isreal, Chelse Spinner, Lisa Sammons, Daniel Fesperman, Racquel Washington, Shivani Mehta, Candace Howell, Jennifer Stamp, Henry Bundy, Pamela Cobb, Bridges Community Partner Group, Audrey Ray

**Affiliations:** 1 Center for Health System Sciences, Atrium Health, 1300 Scott Ave, Charlotte, NC, 28204, United States, 1 7043556562, 1 7043556077; 2 Department of Social Sciences and Health Policy, Wake Forest University School of Medicine, Winston Salem, NC, United States; 3 Department of Epidemiology and Community Health, University of North Carolina at Charlotte, Charlotte, NC, United States; 4 Department of Pediatrics, Stanford Medicine, Stanford, CA, United States; 5 Mecklenburg County Health Department, Mecklenburg County Government, Charlotte, NC, United States; 6 Division on Community Health, Advocate Health, Charlotte, NC, United States; 7 The Queen's Collective Birthing, Charlotte, NC, United States; 8 Department of Pediatrics, Levine Children’s Hospital, Atrium Health, Charlotte, NC, United States; 9 Smart Start of Mecklenburg County, Charlotte, NC, United States; 10 Department of Obstetrics and Gynecology, Atrium Health, Charlotte, NC, United States; 11 See Acknowledgments

**Keywords:** perinatal care, patient education, health disparities, social determinants of health, community partnership, maternal health, infant health

## Abstract

**Background:**

In the United States, Black mothers and their infants experience higher rates of maternal and infant mortality than other racial or ethnic groups. North Carolina mirrors national trends with worse perinatal outcomes for Black families compared with other groups. Most ongoing efforts to address these disparities focus on policy and systems change. Few initiatives focus on education and resource navigation for families.

**Objective:**

This study aimed to design an online resource hub to provide information to support timely access to care and resources to improve perinatal health outcomes for Black families in Mecklenburg County, the largest metropolitan area in North Carolina.

**Methods:**

We used an iterative community-informed process, including focus groups and meetings, to develop and refine the layout and content of an informational website. We conducted focus groups during 2022 and 2023 with Black mothers (n=14) who had given birth in the prior 2 years or were pregnant. A semistructured interview guide explored participant perspectives on (1) information that would be most helpful during the perinatal period, (2) website usability and content, (3) appropriateness of imagery and topics, and (4) effective dissemination strategies. Additionally, the research team met regularly with a multisector community partner group to get feedback on website iterations and solicit community resources to include. All content was reviewed for health literacy. Focus group participants were recruited through local clinics and partnering community-based organizations. Our multisector community partner group included individuals representing public health, patients, providers, social services, and health system leaders. The Reach, Effectiveness, Adoption, Implementation, and Maintenance framework was used for evaluation.

**Results:**

Key themes for website focus areas included (1) vetted information presented in lay terminology, with tools to identify local, affordable, and culturally competent care; (2) information related to the week-to-week changes they could expect during pregnancy; and (3) alternative birthing options. The most common suggestions for improvement related to the navigation, amount of text, color scheme, and the use of images. The final Mecklenburg Birthing Connections resource hub provides educational and informative resources for every stage of the perinatal care journey, from preconception to childcare, and links to community resources to address health care and social needs. Results from outreach and marketing efforts to increase awareness of the resource within the community had a broad reach. In-person events attracted >800 community members and social media marketing engaged >145,000 unique accounts. Focus group discussions revealed that Black mothers feel that the website addresses important informational needs for Black families.

**Conclusions:**

Partnership with community members enabled the design of a tailored online tool for providing timely information to educate and empower Black families. Ongoing maintenance and dissemination may help address local inequities in perinatal health outcomes.

## Introduction

Maternal and infant mortality rates in the United States far exceed those of other high-income countries. In the United States, more than 700 women die of pregnancy-related complications each year, and approximately 50,000 women experience severe maternal morbidity [1]. In 2022, there were 22.3 maternal deaths per 100,000 live births, representing 817 women who died from maternal causes [1]. Infant mortality rates are on the rise, increasing from 5.4 deaths per 1000 live births in 2021 to 5.6 deaths per 1000 live births in 2022 [2]. Underpinning these stark statistics are persistent racial and ethnic disparities that have yet to be comprehensively addressed by the US health care system. Black and American Indian and Alaska Native (AIAN) women are more than 2-3 times more likely to die from pregnancy-related complications [1]. Black, AIAN, and Native Hawaiian and Other Pacific Islander experience higher preterm births, low birthweight births, or births where they receive inadequate or no prenatal care [3]. Consequently, infants born to Black, AIAN, and Native Hawaiian and Other Pacific Islander women have higher mortality rates than their White counterparts [3].

Several strategies have been proposed to mitigate disparities in maternal and infant health. Ongoing policy initiatives are aimed at the promotion of expanded access to prenatal and postpartum care and coverage for Medicaid beneficiaries who are disproportionately Black, Hispanic, or AIAN [4]. Other efforts have focused on improving the racial diversity of the maternal health workforce to match that of the population [5,6]. Community-based programs such as Healthy Start [7,8] that offer care coordination and service referrals, and home visiting programs [9,10] are other promising strategies. In addition to addressing these structural determinants, there remains a need for person-centered and community-centered approaches that equip and empower families with information to navigate resources available to support their perinatal care needs.

Digital media tools, including websites, mobile apps, SMS, or discussion forums, have been shown to reduce barriers to traditional forms of perinatal care and increase accessibility [11]. For example, digital tools have been used to provide educational information regarding reproductive life planning, postpartum care, anxiety reduction, and health management [12-16]. In a systematic review of 55 studies of digital patient education interventions in the perinatal period, more than two-thirds of studies (n=38) reported a significant positive outcome related to improved patient knowledge, emotional benefit, or improved health behaviors. These tools have the potential to complement other efforts aimed at reducing maternal and infant health disparities by increasing patient empowerment and self-efficacy, supporting patient-provider relationships, and strengthening connections to community resources that lead to improved health outcomes [17,18].

Perinatal populations have indicated a preference for digital health resources that highlight what is typical to expect during pregnancy, provide access to a provider when needed, and offer information on fetal development [19]. Another important consideration in the development of perinatal digital health tools is the quality of health information hosted on these platforms. A scoping review of 30 studies by Li et al [20] concluded that perinatal women were dissatisfied with the quality of maternal and infant health information available on digital media, raising skepticism about the credibility of those delivering the information, the irrelevant or repetitive content amid an overwhelming amount of information, and the lack of scientific evidence. Platform usability and commercialization of digital media platforms have also been criticized by perinatal women using digital media platforms for health information [20].

The increased prevalence and use of digital media provide an opportunity to deliver perinatal health information directly to Black families in health care and community settings. Approximately 87% of Black adults own a smartphone and 94% use the internet [21]. Yet, few digital tools are tailored to the needs of Black people. There is scarce research on the development and testing of digital tools specifically designed to address maternal and infant health disparities among Black birthing people. One study testing the effect of a culturally tailored conversational agent for preconception health education among 528 Black women from 35 US states found a 16% increase in action to improve preconception health risks among intervention versus control participants [22]. Exploring different avenues for digital health research among historically underrepresented perinatal populations is critical as the field moves forward. Research on tailored digital tools that span the life course may further enhance efforts to improve disparate perinatal outcomes.

Guided by the literature, we designed a health system–hosted online resource hub to raise awareness, educate, and empower Black birthing people and their families. The online resource hub was an output of a larger project called the Perinatal Access to Care Campaign, which aimed to provide information to support timely access to care and resources to improve perinatal health outcomes for Black families. This paper describes the collaborative and iterative design process and early evaluation of the online resource hub and provides recommendations for future research and implementation.

## Methods

### Overview

We followed a community-engaged participatory action research approach. Community engagement is defined as “the process of working collaboratively with and through groups of people affiliated by geographic proximity, special interest, or similar situations to address issues affecting the well-being of those people” [23]. To that end, we used a collaborative approach guided by an established multisector partnership group, the Bridges Community Partner Group, which served as a steering committee across all phases of project design and execution. The Bridges Community Partner Group prioritized informational resources as a need for the community and participated in the curation of content and resources.

### Setting and Participants

This project was conducted in Mecklenburg County, North Carolina, a large metropolitan area with more than 1 million residents. The US Census indicates that 33% (383,645/1,163,701) of residents identified as Black or African American in 2023 [24]. Racial and ethnic disparities in infant mortality in Mecklenburg County mirror state and national trends, with Black infants 2.6 times more likely to die before their first birthday than White infants in 2021 (12.6 vs 4.8 deaths per 1000 live births) [25]. At the state level, the North Carolina Perinatal Health Strategic Plan provides guidance to improve maternal and infant health by addressing social and economic inequities, strengthening families and communities, and improving health care for all people of reproductive age [26]. Within the county, Mecklenburg County Public Health leads initiatives to improve maternal and child health, including home visiting programs, care management, and community health worker programs [27]. Atrium Health is a large not-for-profit health care system headquartered in Mecklenburg County, which delivers the full spectrum of care, including maternity care and pediatric care, through its hospital and outpatient clinic locations. Atrium Health extends care into the community through a variety of programs and partnerships [28]. This project was led by a multidisciplinary team of investigators from Atrium Health in partnership with collaborators from Mecklenburg County Public Health and the University of North Carolina at Charlotte.

Members of the Bridges Community Partner Group were identified through a snowball approach. Snowball sampling is a method used to identify individuals from a specific target population and involves starting with a small initial group and then using referrals to identify additional group members [29,30]. Beginning with an initial group of members from departments across Atrium Health, the group was expanded to include additional internal and external partners and community members to ensure representation of diverse perspectives including birthing professions, public health, social services, community, patient, provider, health system, and research, with a final membership of 42. From August 2022 through August 2024, monthly meetings were scheduled virtually for 1 hour in the afternoon to provide the group with project updates and seek input from partners. All meetings began with a reflection on current news and events related to perinatal health to build common ground around the group’s work. Meetings included discussions on the progress of the project and perspectives on the campaign development from the initial website wireframe through the marketing strategy for disseminating the resource.

### Ethical Considerations

The Wake Forest University School of Medicine Institutional Review Board reviewed this project and determined that it did not meet the federal definition of research involving human participants, with no need for continuing review (IRB00087620). Verbal consent was obtained from all focus group participants prior to data collection. Focus group participants received lunch (in-person sessions only) and were offered a US $50 prepaid debit card as compensation for their time. Survey data were collected anonymously, with an acknowledgment that participation was voluntary and that data would be reported in aggregate. No compensation was provided for survey data collection. All data were stored without identifying information to preserve privacy and confidentiality.

### Conceptual Frameworks

We used a life course perspective to guide project design. The life course perspective posits that observed health and health outcomes are the cumulative result of experiences that happen prior to birth and throughout the lifespan [31]. In the case of Black infants, a life course approach highlights the importance of accessing health care across the life course, investing in community building, and closing the education gap, among other strategies [32]. Extending this perspective, we focused on providing education and resources that spanned preconception and prenatal care in addition to delivery, postpartum, and early childcare experiences. The racism as a root cause framework [33], a solutions-focused approach for addressing racial health disparities, also informed our approach. Using this framework, we devised strategies to address the framework’s 4 domains with a specific focus on long-term impact (Textbox 1).

Textbox 1. **Applying racism as a root cause framework to the development of an online resource hub.**

Strategies applied for each domain of the racism as a root cause framework:

- Precise impact 
  - Target Black mothers and caregivers.
  - Identify target zip codes with >50% Black, >9% low birth weight (county median), and median age of <40 years.
- Systems change
  - Develop internal messaging and provider-facing communication about campaign.
  - Connect with decision makers regarding current policies about where women can go for care.
- Long-term
  - Develop a website with information that can be updated beyond the campaign.
  - Create a plan for keeping information current.
- Reparations 
  - Invite Black mothers in target communities to focus groups and pay them for their time.
  - Use feedback from Black community members to guide approach (how, what, and where).

### Iterative Website Development

There were several iterations of the website content beginning with the wireframe, or alpha scripts, in March 2023. The evolution of the website was grouped into 4 major stages (alpha, beta 2.0, beta 3.0, and beta 4.0) representing major transitions in website design and content curation over an 18-month period. Feedback from focus groups and our community partner group informed each stage of iteration.

#### Focus Groups

Across the study period, we held 5 focus groups with community members to gather information on the content that would be most useful to include on the website, usability of the design, and feedback to guide development through the various iterations. Focus group participants were recruited from community organizations located in target zip codes and through associations with the Bridges Community Partner Group. Residents of Mecklenburg County were eligible to participate in focus groups if they identified as Black or African American, were aged 18 years or older, were pregnant at the time of participation, or had a baby in the past 2 years.

Four focus groups were held in person at locations in the community (a local recreation center, a local library, and a residential care organization for expectant mothers). One session was held via videoconference. The first 2 focus groups were held in December 2022 (prior to initial website prototype), and subsequent sessions occurred in August, September, and October 2023. A semistructured interview guide developed based on literature review and input from our community partner group explored participant perspectives on (1) information that would be most helpful during the perinatal period, (2) website usability and content, (3) appropriateness of imagery and topics, and (4) effective dissemination strategies. A total of 14 Black mothers participated across all sessions, with some attending up to 2 sessions. An informed consent document was reviewed with participants prior to the start of each focus group, and verbal consent was obtained from all participants. Focus groups lasted approximately 1 hour and were recorded and transcribed. Focus groups were designed to get iterative feedback on the development of the resource hub. Data collection continued until no new themes emerged. Thematic analysis was used to identify key themes and recommendations across sessions to inform the iterations of website build. Three members of the study team (HB, MI, and YT) led data analysis, and discrepancies were resolved via discussion among all study team members.

#### Community Partner Meetings

In addition to collecting feedback from focus groups with Black mothers, the Bridges Community Partner Group and the project team also contributed feedback to guide the development of the website in a series of iterations. The Bridges Community Partner Group met once a month virtually to review project updates and solicit input on content and delivery of information for the online resource. Biweekly, the project team met to review partner feedback, identify additional resources based on that feedback, and discuss the organization of the information on the website. Changes to the copy, organization of content, or design were collated by a project manager, who organized information into a script for the health system’s website development team to implement.

#### Language and Health Literacy

All website content was framed using a strengths-based approach. Strengths-based approaches are increasingly used in health interventions to give agency and empower the target audience to take action to achieve their goals for health and well-being [34]. We incorporated language such as “I want” rather than “I need” and imagery that appropriately reflected the target audience. It was important for the website to be relatable to community members to encourage connection and relevance to their own care planning. Content was also reviewed for health literacy to ensure accessibility to a broad audience.

### Marketing and Outreach Campaign

We collaborated with the health system marketing team to design and implement a marketing and outreach campaign for the website. We used the Charlotte-Mecklenburg Quality of Life Explorer [35] to identify target neighborhoods with >50% Black population, median age <40 years, population size >1000, and low birth weight rate >9% (the county overall rate). We identified 9 neighborhoods across 4 zip codes (28205, 28206, 28208, and 28217) that became targets for marketing and outreach following review and input from our community partner group. To assist our outreach efforts, the marketing team developed flyers in both English and Spanish for distribution at community events and clinic locations and via community organizations and partners. The marketing team also developed a campaign that ran from August to October 2024 and included advertisements through the Atrium Health social media channels on Instagram and Facebook as well as out-of-home marketing (billboards) across the target zip codes.

### Evaluation Framework

We used the Reach, Effectiveness, Adoption, Implementation, and Maintenance evaluation framework [36] to assess the early impact of the online resource hub and our marketing and outreach efforts. Reach was measured using website and social media statistics, along with attendance at community events. Website traffic was captured by unique visit data and QR code scan totals driving traffic to the site. Social media metrics included impressions, link clicks, likes, and shares. Billboard impressions, a measure of actual and potential ad views based on traffic patterns, were provided by our marketing partner. At community events, attendance was used as an indicator of website reach, as attendees were informed about the resource or received promotional materials. Effectiveness was measured using qualitative feedback from community members. Adoption and implementation in the short term were assessed with an anonymous survey sent to the Bridges Community Partner Group. The 10-item online survey assessed partners’ perspectives on whether the campaign met the intended goal, overall satisfaction with the website, and the website usability, readability, accessibility, and cultural relevance. In addition, partners were asked to report how they were disseminating the resource to their personal and professional networks. As part of the final email to thank the partner group for their effort on the project, a request to participate in the feedback survey was embedded in the email with a response window of 2 weeks. A single reminder was sent. This process feedback collected voluntarily did not constitute human participants research or require institutional review board review. Survey results were reported as counts and percentages. Finally, maintenance was assessed by our ability to develop a plan for maintaining and updating website content and integrating it into routine health system workflows.

## Results

### Key Themes From Focus Groups and Community Partner Meetings

Themes from initial focus groups informing the website design are included in Table 1. Black mothers participating in the focus groups shared what they would like to see in an informational resource. Key themes for focus areas included vetted information presented in lay terminology, with tools to identify local, affordable, culturally competent care, information related to week-to-week changes to expect during your pregnancy, and alternatives to the care options of the hospital. Participants favored having information in a mobile app but were open to having the information in a mobile-friendly website, which was the most feasible option for the project. These findings were reviewed with community partners who agreed with these focus areas and added content for caregivers and connections to community resources.

**Table 1.** Themes from focus groups with Black mothers to inform initial website prototype. Participants were asked about their informational needs and what to include in a resource for Black families.

Theme	Representative quote
Have vetted and distilled information, as to not overwhelm.	“You don’t want to go too crazy on the Internet. Because I have tried to diagnose myself with certain stuff, and you’ll get scared.”
Include current and updated information.	“With my third daughter, it had been a while, and I had nursed, but I needed a refresher.”
Have a way to save pregnancy-related details.	“I can’t keep up with that stuff [scheduling regular specialist appointments].”
Include personalized recommendations.	“It was like, they read a book. And they thought that book applied to every patient.”
Have information on what to expect, week-to-week, during your pregnancy.	“Being able to map it out, so that we can mentally be prepared.”
Answer basic questions about pregnancy.	“There was this assumption that I should know. But it’s my first time. Why would I know?”
Provide information on sources of culturally competent care.	“I was literally calling saying, hey, do you have a doctor that looks like me?”
Foster community and allow for the sharing of birthing/pregnancy experiences.	“If no one does it for us, we have to be able to seek out things. You need to know who you express these concerns to along the way.”
Discuss and explain affordable alternatives to the care options of the hospital.	“I felt like the only option they were trying to give was medicine.”
Be comprehensive.	“...I was given an app…but I will say, interface-wise, compared to What to Expect and all that, I don’t like the app. So, I don’t even really use it because it wasn’t as user-friendly and didn’t have as much information…it would have made a difference for me to have all [the information] in one place. Because I wanted to like the one they gave me. The doctor said I should download and keep up with it, but I didn’t like it. It didn’t have all the things I felt it needed to have.”

Black mothers attending focus groups where drafts of the website were reviewed had a positive initial reaction to the organization of the website, finding the organization and esthetic clear, and the naming communicative of the site’s purpose. The most common suggestions for improvement related to the navigation, amount of text, color scheme, and the use of images. Focus group participants expressed shock at the statistic on the home page, which revealed that Black infants were twice as likely to die as White infants in Mecklenburg County. This topic came up often in feedback, with community partners recommending a “focus on joy” on the home page along with the benefits of the website. Community members still found the statistics informative and recommended that they appear less prominently on the page. Mothers found the use of images containing Black women and babies comforting and familiar, making the information personal to them. Additional feedback included providing more information on topics such as insurance, healing milestones in the postpartum period, lactation, pregnancy symptoms and complications to be aware of, resources for dads, honoring the loss of a child, and changing family dynamics.

### Iterative Website Evolution

**Figure 1.**
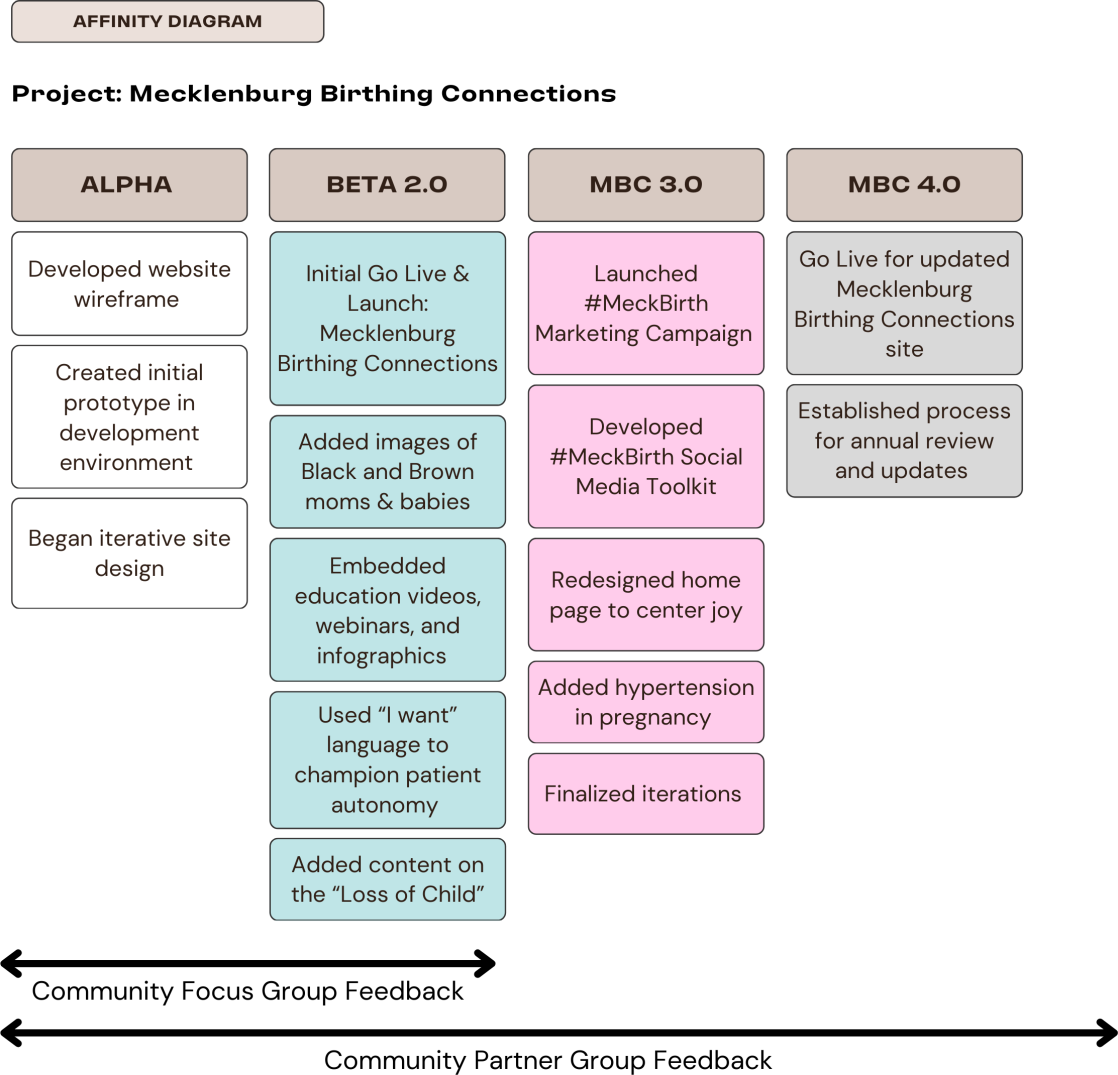
Stages of development of the Mecklenburg Birthing Connections online resource hub.

Major stages of website development are outlined in Figure 1. In the alpha stage of development, the website wireframe began as a PowerPoint slide deck that provided a general layout of the website content with sections for information on preconception care, pregnancy, postpartum, early childcare, and community resources as informed by focus groups and meetings with community partners. Once the general layout was approved by our community partner group, we started to curate content in a document with headers, text, and links. Content was curated from reputable websites such as the Centers for Disease Control and Prevention, American College of Obstetricians and Gynecologists, and from experts within our community partner group. We shared the copy of the curated content with the Atrium Health Marketing and Web Design team, who then created the first website prototype in a development environment behind the health system firewall.

The beta 2.0 stage included additions to the resource beginning with the selection of a website name, Mecklenburg Birthing Connections, which was voted on by our community partner group. We built out linkages to the health system’s community resource hub, a searchable database of community resources by zip code, for topics including housing, childcare, and food. We also developed and added 27 infographics highlighting select website content in a brief, visually pleasing format. Other modifications during this stage included adding introductory text with header images to each page and reviewing website language to ensure alignment with goals for empowering our target audience. New topic areas added included elective abortion, miscarriage, and induction. This phase of development included iterations for content, text, or format and concluded in April 2024 with the official go-live of the public-facing website [37]. This marked the end of the primary content development phase. To celebrate the official launch of Mecklenburg Birthing Connections, the project team hosted a community forum and resource fair to bring awareness to the newly launched resource, collect feedback, and connect community members to local organizations serving families during the perinatal period. This also marked the beginning of grassroots community outreach to increase awareness of the resource within the community.

The 3.0 stage had a focus on dissemination and expanding partnerships. Our grassroots approach involved identifying and participating in local community events focused on maternal, child, and family well-being. We prioritized events that focused on Black families and events that focused on families with low incomes. Events included community resource fairs, health fairs, conferences, walks, runs, and other virtual speaking opportunities within the health system (ie, local provider meetings) and the local community. We used this approach to reach people living in the community and those serving in health care and social service organizations. A central output of our outreach activities was identifying new partners and resources to add to the website and expand our reach. For example, the project team partnered with local doulas and a local university to cosponsor a webinar titled, “Black Birthing Matters” on the role of doulas, midwives, and obstetricians in the birthing room. The webinar recording was subsequently added to the website as a resource. Another collaboration with a local social service organization to cohost an event focused on fathers also provided an opportunity to solicit feedback from fathers on the content of the online resource. Other modifications to the website at this stage included a redesign of the home page, addition of a social media tool kit for sharing with community partners, and a collaboration with another health system initiative to build out content on hypertension in pregnancy, which disproportionately affects Black mothers.

The 4.0 stage marked the final phase of development. We obtained sign-off from our partners and closed the website for major revisions. The final Mecklenburg Birthing Connections website includes educational and informative resources for every stage of the perinatal care journey, from preconception to childcare, and links to a searchable database of community resources to address health care and social needs. In addition, downloadable infographics highlighted vital website content in a graphically engaging format.

### Marketing and Outreach Campaign

#### Reach

Community outreach events are outlined in Table 2. Across 13 events between March and October 2024, we reached more than 800 community members. Events included conferences, networking meetings, webinars, and community forums. In addition, we delivered 1170 flyers and rack cards across 9 clinic and community locations for further dissemination.

**Table 2.** Community outreach activities.

Event title	Date	Type of event	Host	Numerical reach, n
Atrium Health Nurses Meeting	March 27, 2024	Clinical Leaders Meeting	Atrium Health	60+
MBC^a^ Community Forum	April 17, 2024	Community Outreach	MBC Project Team	60+
Community Health Worker- Resource Fair	April 20, 2024	Community Outreach	Atrium Health	150+
Black Maternal Health Conference	April 11, 2024	Conference	Care Ring	100+
Black Maternal Health Week Walk	April 20, 2024	Walk/Run	Care Ring and BirthRight	30
Regional Breastfeeding Conference: 25th Annual Mother’s Special Gift Breastfeeding Conference: We’ve Come a Long Way Baby!	April 22, 2024	Conference	Area Health Education Center	30
Public Health Lunch & Learn Webinar	May 22, 2024	Webinar	Mecklenburg County Public Health	10
Queen City Community Connect Coalition- Q2 Meeting	June 4, 2024	Networking and Advocacy for Community Health Workers	Queen City Community Connect Coalition	30
Superman & Me: Celebrating Fatherhood	June 22, 2024	Community Outreach	MBC Project Team and Smart Start	15
Meck Pre-K Orientation	July 20, 2024	Orientation for new Pre-K families	Smart Start	60
Hey Moms, Hey Dads	August 3, 2024	Community Outreach	Department of Health and Human Services	150+
Black Birthing Matters	September 20, 2024	Webinar	MBC Project Team and Charlotte MCH^b^ Workgroup	136
Jace’s Journey 5k Run/Walk	October 26, 2024	Walk/Run	Jace’s Journey	50+

a MBC, Mecklenburg Birthing Connections.

b MCH, Maternal Child Health.

**Figure 2.**
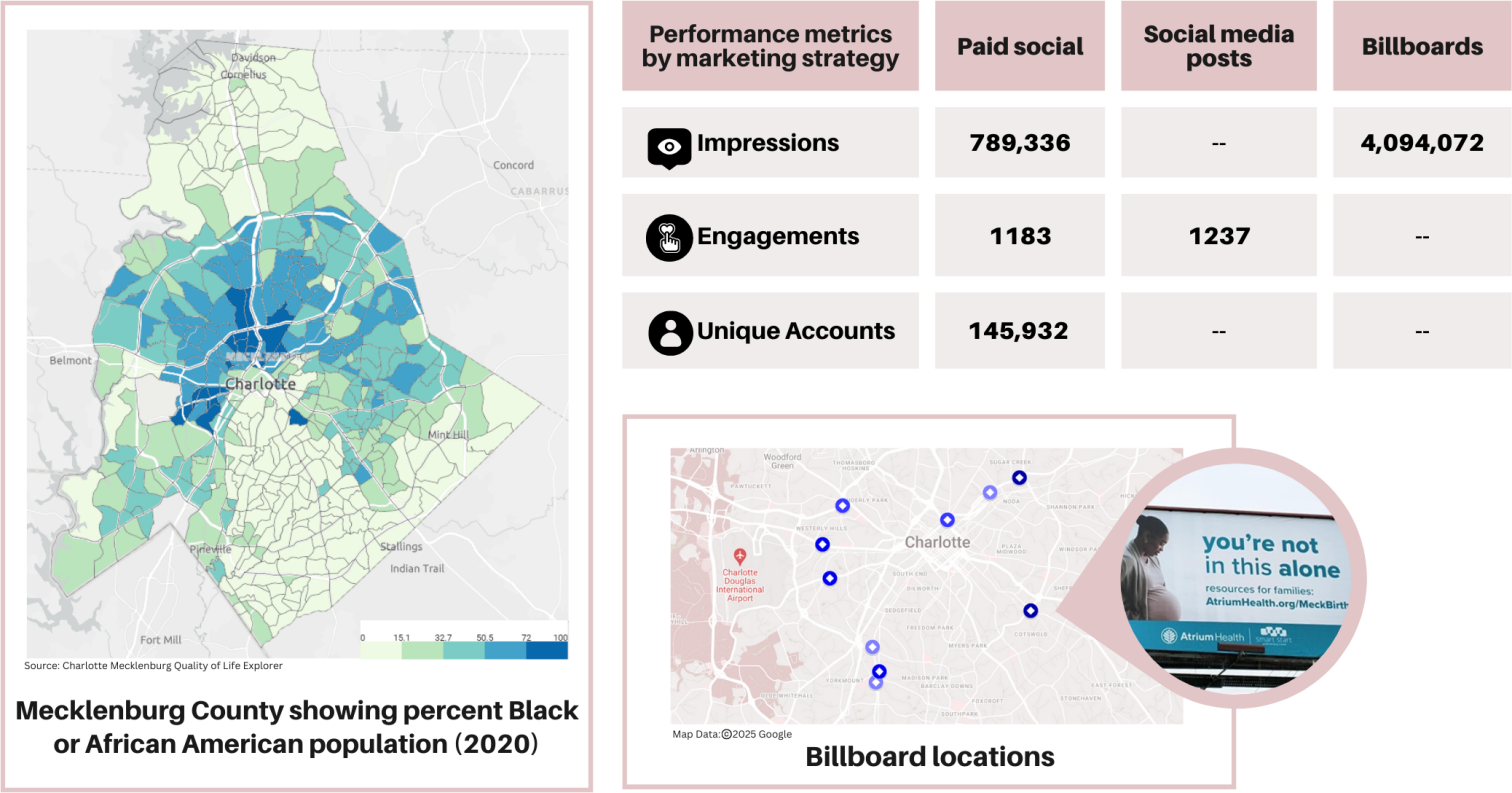
Reach of the Mecklenburg Birthing Connections marketing campaign.

Marketing campaign results are summarized in Figure 2. Paid social advertising reached 145,932 unique accounts and resulted in 789,336 impressions (the number of times individuals were exposed to ads) and 1183 link clicks. Engagement with posts on social media reached 1237. Billboard placement was optimized to high-traffic street posters in select zip codes to drive high impressions efficiently. Billboards were placed for 6 weeks from August 12 to September 22, 2024, with a total reach of 4,094,072 impressions (number of times individuals were exposed to billboards) across 10 locations.

#### Effectiveness

Qualitative feedback from community members captured website effectiveness regarding goals for providing content that met the needs of Black families in a format that was accessible. Major themes included informational gaps for Black families, clarity and organization of content, and practical use and accessibility. Regarding informational gaps, community members found the website informational and helpful for answering questions that are not typically addressed elsewhere. One focus group participant commented, “Yeah, I think it’s very informational. It’s very detailed. It actually answers a lotta questions that a lot of us may have that aren’t given on the regular website.” And another shared, “I was surprised that y’all had postpartum depression because it’s not really talked about in the Black community a lot. That’s a good asset as well.” With respect to clarity and organization, community members shared positive feedback about the structure of the website. A focus group participant commented:

*Feel like it was structured pretty well. It broke it down, the stages of pregnancy, like before. Some people think about it. Some people don’t. When you get pregnant, the resources that you can do while you’re pregnant to prepare for the pregnancy, and then after the pregnancy. You even catered to those that might have a stillbirth or a miscarriage or somethin’ of that sort*.

Community members also shared thoughts on the practical use and accessibility of the website. When asked about how they would use the information, 1 focus group participant responded, “I think I would use this if I was pregnant ’cause it gives you, pretty much, every step. It walks you through most of the pregnancy and the whole being pregnant, after pregnant. I would definitely look into this if I was just pregnant.” Another commented:

*I mean, it’s very helpful...I can just come here and get the information, and I can pinpoint exactly what I’m looking for, it’s very helpful. I haven’t come across anything where I can say, ‘Oh. If I can look at this...’ If I can go through the information and all in one place, it would be very helpful for me. Yeah. I could even pass it along to anybody else*.

Other themes included inclusivity and representation and community connection and peer support. Community members felt that the website used imagery effectively to represent diverse Black women and recommended more explicit focus on fathers and extended family members. Community members also expressed interest in being able to connect with other parents via support groups and meetups and identified that as an area that the resource hub could improve upon.

#### Adoption and Implementation

Feedback from the Bridges Community Partner Group was requested following the launch of Mecklenburg Birthing Connections. Six community partners responded to an anonymous online evaluation survey exploring satisfaction with the website and adoption and implementation of the resource. Unanimously, the respondents felt that the goals of the project to provide targeted communication to prioritized communities with poor outcomes were met “to a great extent” (6/6, 100%). Feedback about meeting the goals included, “I believe that the website has become a comprehensive place with information from a variety of partners to represent a variety of resources,” and “The focus was targeted to Black mothers and Black birthing parents. The information shared was very specific and with a focused aim.” Partners’ (n=6) average satisfaction with the overall experience on the website was 8.67 out of 10 (SD 1.21). Website navigation was rated an average of 8.83 out of 10 (SD 0.75). Partners (n=6) assessed the readability of information on the website with an average of 9.5 out of 10 (SD 0.84). Most partners believe that the website meets the needs of their average client regarding cultural resonance (5/6, 83%), readability (5/6, 83%), and accessibility (4/6, 66%). The other respondents indicated these factors to be “somewhat met.”

With the recent launch of the website, partners were asked to share how they have implemented the website within their organization. The majority shared the website with a colleague (4/6, 67%) or provided clients with a direct referral link (3/6, 50%). Two of the 6 (33%) respondents promoted the link in email distributions (eg, newsletters). Only 1 out of 6 respondents (17%) indicated the use of printed materials to promote the website within their organization. When exploring resource distribution outside of their organizations, partners described sharing the direct link with friends and family.

#### Maintenance

Maintenance of the online resource hub as a tool to promote positive perinatal health outcomes for Black families focused on integration with health system workflows and connections to community. Within the health system, the Women’s Care Service Line, which includes oversight of the Department of Obstetrics and Gynecology, was identified as a primary avenue for ongoing dissemination. In addition, we engaged the health system’s Community Health Worker team for ongoing dissemination among community members. The plan for regular updates to the online resource hub included adding a feedback link that will be reviewed annually by the project team to prioritize updates and reviews. Future evaluation of maintenance will include tracking visits to the site and gathering input from partners regarding their use of the resource in the community.

## Discussion

### Principal Findings

Improving maternal and infant outcomes for Black mothers and babies in the United States is an urgent priority [38]. However, many efforts do not fully address the impact of social, economic, or environmental factors that contribute to disparities in maternal and infant health [39], and few focus on education and empowerment for families. Our efforts to develop a community-informed informational resource addressed an important gap by incorporating diverse community perspectives and focusing on the continuum from preconception health to early childhood. Moreover, the integration of this resource within a local health system lent support to ongoing efforts to engage health care providers in addressing social determinants of health, while providing a path for ongoing use and dissemination of the tool. Our focus on the Black birthing experience and patient, family, and caregiver empowerment is an additional unique contribution that community members found useful and needed.

Our work aligns with other similar efforts to address maternal and child health disparities using community-engaged approaches, as recommended by the National Institutes of Health [40]. For example, the Commonwealth Fund supported the development of the Maternal Health Hub operated by the Health Care Transformation Task Force [41]. The Maternal Health Hub is an online resource that contains resources and best practices to enhance health equity, increase value-based health care payment models, and promote public policy to reduce maternal health disparities. Furthermore, the National Institutes of Health Office of Research on Women’s Health created a Maternal Morbidity and Mortality Web Portal providing a central hub of information regarding the maternal health crisis.

Similar to our approach of involving vested individuals in the curation of materials, Bower et al [42] describe the development of a Maternal Health Equity Toolkit in Maryland by a work group comprising health disparities researchers, hospital-based improvement specialists, perinatal health providers, and representatives of community-based organizations. Each chapter of the toolkit was reviewed by at least 2 experts, with feedback strengthening the use of inclusive language, logical sequencing of strategies, and action steps. The final product included the engagement of locally based resources by linking patients to community and social services.

Despite comprehensive searches, few papers describe educational websites dedicated to Black maternal health education. Most research focuses on the use of mHealth and social media to provide education, which differs significantly from Mecklenburg Birthing Connections [11,43-45]. A study by McFarlane et al [46] describes the pilot evaluation of a multimedia childbirth education intervention for Black women and their birth companions. The intervention, which involved animations and games that were designed in consultation with a Black doula organization and experts in obstetrics and gynecology, was found to be feasible and acceptable for education on Black maternal health disparities, with moderate impacts on self-efficacy and agency [46]. This study differed from ours in that it was not accompanied by a full website platform and had a specific focus on social support in the hospital setting. However, it provides additional evidence for the need for culturally tailored birthing education for Black families, feasibility of web-based tools, and the benefit of co-design when developing such interventions.

### Health System–Community Partnership

The health system–community partnership model used to develop the online resource offered benefits as well as challenges to navigate. Benefits included access to health system resources for web development and marketing expertise as well as a platform to engage providers and administrators on the topic of patient education and empowerment to reduce health disparities. This work also aligned with ongoing efforts within the health system’s women’s care service line to advance maternal health equity. For example, the health system had launched a women’s care bus that toured target neighborhoods with limited access to primary and preventive care and was developing a doula program to address poor Black maternal health outcomes. Challenges experienced were due touncertainty in policies and decision-making hierarchy related to the health system’s active mergers and integrations, and shifting timelines due to capacity and prioritization of other projects. This required remaining flexible and maintaining regular communication with health system partners. For example, we had to delay aspects of our marketing plan that depended on health system resources because of competing priorities and begin with grassroots outreach. Ultimately, these opportunities proved to be beneficial in brand recognition and relationship development, not only with families who may use the website but also with the community organizations who hosted or cohosted events with the project team.

Community partnership was invaluable for achieving a product that community members felt reflected their needs. Including community partners from the beginning and engaging community members in decisions regarding what information to include on the resource hub, identification of additional impactful partners in the community, and review of website content during development phases were important aspects of our approach. This required the project team to lean into principles that build trust, such as acknowledging that our community members were experts who we needed to learn from along the way, and building relationships that would extend beyond the time frame of this funded project [47].

### Limitations

Our approach had a few limitations. While we benefited from perspectives of a diverse community partner group that included male and female Black parents and caregivers, we included only Black mothers in our focus groups. Additional data collection from Black fathers in the future would help gather more insights into their informational needs and additional content that would benefit them. The potential for selection bias among focus group participants is another limitation that we were able to mitigate by triangulating focus group findings with community partner feedback. Also, hosting the final website within the domain of an established health care system limited our ability to incorporate resources from organizations identified as competitors. Notwithstanding, we were able to leverage the health system’s broad reach within the local community, its many existing community partnerships, and its position as the largest Medicaid provider in the state. Finally, we were able to capture only the early effectiveness of the resource, and the adoption and implementation results were based on small sample sizes. Future evaluation could examine the impacts of the resource hub on perinatal care experiences of community members and assess the adoption and implementation among a broader sample of community members.

### Implications for Practice and Translation

Our work offers opportunities for future dissemination and translation in clinical and community settings. For example, this online resource could be used to connect to resources where clinics lack embedded social work resources. The user-friendly online format also allows patients to review content at their leisure, thus bridging gaps in timely access to information. Tools like ours that extend beyond the clinical setting to home and community settings have potential for increased impact on reducing racial and ethnic disparities in care and outcomes by considering the social and cultural context in which patients live [48]. Patient empowerment also aids in the reduction of health disparities by giving patients increased agency in their health care decisions [49]. This online resource was designed to equip patients and families to navigate resources in their communities, a crucial component of patient-centered care. The design of Mecklenburg Birthing Connections as a resource hub further supports its ongoing use as a repository for information about programs and services provided by the health system and community partners to support Black families in the future. Other health systems and communities can adapt our approach by soliciting feedback on local perinatal needs and using those findings to tailor resources in collaboration with families and multisector community-based partners. In particular, with the limited and diminishing resources available to support adequate perinatal health in rural areas of the United States [50], developing tailored resource lists for rural families may help supplement care for high-risk populations. Our tool could serve as a foundation for rural counties surrounding the metropolitan area, or within the state, as some of the listed organizations and community partners have multiple locations.

### Conclusions

An online resource hub is a valuable approach for providing timely information to educate and empower Black families and caregivers during the perinatal journey. Community members value having a comprehensive tool that focuses on the perinatal experiences of Black families. Communities seeking to improve access to care for Black families may consider a similar approach to identifying and collating local resources in collaboration with local community members and partners to provide easy access to education and information to address local inequities in perinatal health outcomes. Future efforts should plan implementation evaluations to measure impact.

## Abbreviations

AIAN: American Indian and Alaska Native
